# Unraveling City-Specific Microbial Signatures and Identifying Sample Origins for the Data From CAMDA 2020 Metagenomic Geolocation Challenge

**DOI:** 10.3389/fgene.2021.659650

**Published:** 2021-08-05

**Authors:** Runzhi Zhang, Dorothy Ellis, Alejandro R. Walker, Susmita Datta

**Affiliations:** ^1^Department of Biostatistics, University of Florida, Gainesville, FL, United States; ^2^Department of Oral Biology, University of Florida, Gainesville, FL, United States

**Keywords:** microbiome, OTU, feature selection, random forest, support vector machine, multilayer perceptron

## Abstract

The composition of microbial communities has been known to be location-specific. Investigating the microbial composition across different cities enables us to unravel city-specific microbial signatures and further predict the origin of unknown samples. As part of the CAMDA 2020 Metagenomic Geolocation Challenge, MetaSUB provided the whole genome shotgun (WGS) metagenomics data from samples across 28 cities along with non-microbial city data for 23 of these cities. In our solution to this challenge, we implemented feature selection, normalization, clustering and three methods of machine learning to classify the cities based on their microbial compositions. Of the three methods, multilayer perceptron obtained the best performance with an error rate of 19.60% based on whether the correct city received the highest or second highest number of votes for the test data contained in the main dataset. We then trained the model to predict the origins of samples from the mystery dataset by including these samples with the additional group label of “mystery.” The mystery dataset compromised of samples collected from a subset of the cities in the main dataset as well as samples collected from new cities. For samples from cities that belonged to the main dataset, error rates ranged from 18.18 to 72.7%. For samples from new cities that did not belong to the main dataset, 57.7% of the test samples could be correctly labeled as “mystery” samples. Furthermore, we also predicted some of the non-microbial features for the mystery samples from the cities that did not belong to main dataset to draw inferences and narrow the range of the possible sample origins using a multi-output multilayer perceptron algorithm.

## Introduction

The development of next-generation sequencing (NGS) platforms (Simon and Daniel, 2011) has enabled the generation of high-throughput metagenomics data, which allows researchers to uncover the microbial composition and function of diverse ecosystems (Ley et al., 2006; Hårdeman and Sjöling, 2007; Wu and Sun, 2009). In recent years, microbiome research, especially within the context of human health and disease, has become of increasing interest to the researchers. For instance, the intestinal microbiome has been proven to be associated with obesity, diabetes mellitus type 2, and a broad range of other diseases (Hartstra et al., 2015; Lynch and Pedersen, 2016). However, many of the microbial communities outside the human body remain poorly studied. As the composition of environmental microbial communities is known to be location-specific (Delgado-Baquerizo et al., 2018), investigating the differences in composition of taxa across locations could help unravel city-specific microbial signatures and further identify the origin of unknown samples. Extending this kind of research to a global scale would help identifying the source of possible pathogens responsible for global pandemics such as in the current COVID-19 crisis. In addition, it could enable forensic scientists to verify location information about objects based on analyzing the microbial data of swabs taken from these objects to establish or provide evidence against alibis. Metagenomic data based on soil samples have already been used to discriminate between different locations (Khodakova et al., 2014).

The objective of the CAMDA 2020 metagenomic geolocation challenge was to predict the geographic origins of mystery samples by using city-specific microbial and non-microbial data. All raw data in this work was provided by the MetaSUB Consortium^1^. MetaSUB aims to create a global genetic cartography of urban spaces that is based on extensive sampling of mass-transit systems and other public areas across the globe. They partnered with CAMDA to provide data for one of the three annual CAMDA challenges for 2020. These data include over a thousand novel samples from 28 cities and comprise over a terabase of whole genome shotgun (WGS) metagenomic data. The main dataset included nearly 1000 samples from 23 cities in 17 countries. An additional microbial dataset with 10 cities (5 of which were represented in the main dataset and 5 new cities) was provided as a mystery dataset. In addition to the microbial data, CAMDA also provided some city-specific data related to climate, location, and neighboring biomes for the main dataset.

**FIGURE 1:**
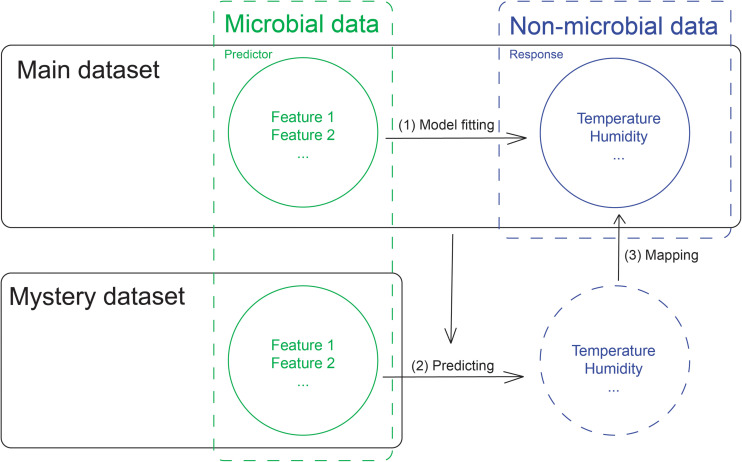
The flowchart for the non-microbial feature prediction for the mystery samples.

Classification methods are typically used to construct models for predicting the origin of a novel sample. For the mystery samples that originated from cities included in the main dataset, we were able to use only 16S ribosomal RNA mapped compositional data to perform classification. One limitation of classification models is that they can never predict a novel origin that does not exist within the trained model. To address this problem, we can use city-specific microbial data to predict non-microbial city-specific features such as temperature and humidity (Figure 1). We can then in turn use these results to map the known city-specific non-microbial patterns, which enables us to narrow the range of possible cities and draw inferences about each pattern.

The selection of the non-microbial features was driven by our knowledge of factors that may possibly affect microbial composition. Microbial community composition and function are altered when microbes are exposed to different climate conditions (Classen et al., 2015). In addition, microbial compositions can vary between coastal and inland locations (Lin et al., 2012) and form distinct groups when populations of microbes are compared across human population densities (Wang et al., 2017). We incorporated various city-specific weather data, a coastal indicator, and neighboring biomes data (a proxy for population density) as output variables for a multi-output multi-layer perceptron (MLP) algorithm (Allaire and Chollet, 2019) based on the main dataset and performed predictions of these for each mystery sample. From these predictions, we could in turn narrow the range of possible origins for samples from cities that were not included in the main dataset. Using climate information as the classification output would enable us to obtain a location-based fingerprint for cities that are not included in the training dataset so long as the climate of the new cities resembled the climate of the cities included in the training dataset.

After bioinformatics and data preprocessing, the operational taxonomic units (OTUs) were aggregated as counts. The selected taxonomic ranks “order,” “family,” and “species” were used independently for the analysis. Table 1 presents a tabulated insight of the data for all the cities from the main dataset. For some of the cities in the main dataset, their samples were collected in multiple years. However, longitudinal analysis was not ideal for this dataset since most of the cities were sampled only once. Being aware of the fact that the microbial compositions of these samples may not be independent and could be similar, we still treated samples collected in different years as being from different cities to account for technical variability such as batch effects. For the multi-output MLP model, since climate does not change significantly from year to year, we did not differentiate between years and only labeled the data based on their climate information; samples from the same city within different years would be trained with the same output labels for the multi-output MLP.

**TABLE 1:** Details of the cities included in the main dataset.

City	Country	Year	Species	Family	Order
Stockholm (ARN)	Sweden	2017	308	122	65
Barcelona (BCN)	Spain	2016	389	123	61
Berlin (BER)	Germany	2016	526	178	98
Denver (DEN)	United States	2016	65	30	21
		2017	143	81	41
Doha (DOH)	Qatar	2016	284	114	58
		2017	249	99	47
Fairbanks (FAI)	United States	2016	457	137	78
Hong Kong (HKG)	China	2017	489	151	82
Seoul (ICN)	South Korea	2017	327	122	64
Kiev (IEV)	Ukraine	2017	426	130	66
Ilorin (ILR)	Nigeria	2016	305	69	34
		2017	628	192	111
Kuala Lumpur (KUL)	Malaysia	2017	191	87	51
London (LCY)	United Kingdom	2017	381	131	63
Lisbon (LIS)	Portugal	2016	196	47	23
New York (NYC)	United States	2016	558	149	75
		2017	649	173	86
Offa (OFF)	Nigeria	2016	387	91	47
Sao Paulo (SAO)	Brazil	2017	245	102	56
Santiago (SCL)	Chile	2016	639	188	107
Sendai (SDJ)	Japan	2017	307	113	69
San Francisco (SFO)	United States	2017	168	86	46
Singapore (SGP)	Singapore	2017	581	171	98
Taipei (TPE)	China	2017	549	181	114
Tokyo (TYO)	Japan	2016	589	177	100
		2017	461	143	78
Zurich (ZRH)	Switzerland	2017	353	124	64
All cities	–	–	1,394	288	193

## Materials and Methods

The design of the analysis was motivated by our previous CAMDA experiences (Walker et al., 2018; Walker and Datta, 2019; Zhang et al., 2021). In our previous work, the common features that existed in all cities proved to provide an effective microbial fingerprint for predicting sample origins, and the use of a combination of common features from different taxonomic ranks rather than only using a single taxonomic rank resulted in the lowest error rate for predictions. Compared to the 2019 MetaSUB challenge with 302 samples from 16 cities, the main dataset for 2020 comprised more than 1,000 samples from 23 cities with samples from some cities collected over multiple years. Since more cities were included, the features that were common to all the cities were more limited. To address this problem and obtain an appropriate number of common features to use for the prediction, we first conducted a less conservative common features selection to serve as a preliminary filter. Then we implemented a variable selection method to further select relevant features for prediction and obtained different feature sets by setting different cutoffs. We then used supervised techniques to select the feature set with the lowest classification error rate. We also used this selected feature set to predict the non-microbial city-specific features. All analyses in this study were conducted in R. The R codes for the analyses will be available on https://susmdatta.github.io/software.html. We provide a more detailed description of the implementations in the following sections.

### Bioinformatics and Data Processing

Samples from MetaSUB were Illumina-sequenced at different depths and delivered in FASTQ format. The bioinformatics and data processing were conducted in the “HiPerGator 2.0” high-performance computing cluster, which includes 50,000 cores with 4GB of RAM in average for each core, at the University of Florida. The run time for each job was highly associated with the size of the raw FASTQ file.

The pipeline utilized in the generation of the OTUs was originally designed and improved in our previous papers (Walker et al., 2018; Walker and Datta, 2019). Using FASTQC (Andrews, 2010) to perform preliminary quality control, we found that the sequencing data were mostly of good quality, with most Phred scores greater than or equal to 38. Based on this result, the Phred score filtering was implemented with FASTX-Toolkit (version 0.0.14 released January 05, 2014) (Patel and Jain, 2012). The parameters used in the filtering were *q* = 38 as the minimum Phred score to keep and *p* = 50 as the minimum percentage of bases with Phred score ≥ 38. We then transformed the quality filtered FASTQ files into FASTA format for performing open reference OTU picking with QIIME (Caporaso et al., 2010). For large samples, we split the FASTA file and parallelized the job into 10 independent sub-threads. After OTU picking in open reference mode, we removed all counts that had a Ribosomal Database Project (RDP) classifier taxonomy assignment score (Wang et al., 2007) of less than 0.5. All subsequent data processing and analyses were then conducted in R (R Core Team, 2018).

### Feature Selection

We used “order,” “family,” and “species” as independent features for all OTUs, “genus” was not used since information about “genus” was included in the “species.” For OTU reaching “genus” resolution but not “species” level, the “species” name would be showed as “*genus.spp*,” for example, *Enhydrobacter.spp*. For each city, there were several samples with extremely low counts when compared to other samples within the same city. These samples contained little information and could hinder the ability of models to predict sample origin. To remove these outliers, we calculated the sum of the count of all orders for each sample and then defined the cutoff for inclusion as 20% of the median of the calculated count within each city. For each city, we removed samples with a calculated count that was less than the cutoff. After this, we conducted preliminary feature selection to obtain the potential features by calculating the ubiquity of features across all the filtered samples, ordering the features by ubiquity, and retaining features with ubiquity >0.6.

Including too many features in the classifier is computationally expensive and runs the risk of overfitting for some of the classifiers we used. To further select the features that were most relevant to city prediction, we used the implementation of elastic net (Zou and Hastie, 2005) logistic regression from the R package *glmnet* (Friedman et al., 2009) to perform variable selection. For each city, e.g., city A, we replaced the labels of all other cities with “not city A.” Since we transformed the city labels of all samples into a binary outcome, we could therefore conduct logistic regression on our dataset. By solving the equation in (1), where *x*_*i*_ is the feature data for sample *i*, *y*_*i*_ is the city indicator mentioned above for sample *i*, β_0_ and β are the coefficients for intercept and all the features, and λ, α are the two parameters controlling the elastic net penalty, we could obtain the regression coefficient for each feature:

$$\min_{\beta_{0},\beta \in R^{p+1}}-\frac{1}{N}\,\sum_{i=1}^{N}\left[y_{i}\,\left(\beta_{0}+x_{i}^{T}\,\beta \right)-l\,o\,g\,\left(1+e^{\left(\beta_{0}+x_{i}^{T}\,\beta \right)}\right)\right]$$

(1) $$+\lambda \,\left[\left(1-\alpha \right)\,{||\beta ||}_{2}^{2}/2+\alpha \,{||\beta ||}_{1}\right]$$

To achieve a balance between feature retention and removal, we manually set the α parameter for the elastic net to 0.4. We calculated the best λ via the cross-validation procedure included in the *glmnet* package and conducted this same procedure for each city within the main dataset. Next, we ordered the features by the number of times the feature was retained by all the models. There were 28 models (23 cities) for the main dataset since some of the cities were with multiple collection years. We used this ordering to determine the cutoff values in downstream analyses. The more times a feature was retained by the models, the more likely the feature was to differentiate the samples of one city from the other cities. By setting different cutoffs, we obtained multiple feature sets that we in turn used for machine learning analysis to obtain the feature set with the best prediction performance. We then used the “voom” function (Law et al., 2014) in the R package *limma* (Ritchie et al., 2015) to normalize the data via log2 counts per million reads to make the data more interpretable while ensuring that counts were bounded away from zero.

### Clustering for the Non-microbial City Data

The non-microbial feature selection was driven by our knowledge of the factors that may possibly affect the microbial composition. As the city-specific data were a combination of continuous and categorical data, to identify patterns in cities’ climate, location, and urbanization, we transformed the continuous data into categorical variables. For the city climate, data were provided in the form of “month-climate-stat,” for example, “July-temperature-minimum.” As cities in the main dataset were from different hemispheres, climate data from the same months corresponded to different meteorological seasons for the southern hemisphere cities and northern hemisphere cities. Since we wanted to categorize the data based on summer and winter temperature and humidity, we needed to transform the original monthly data into the corresponding meteorological season (National Oceanic and Atmospheric Administration, 2016) to make the data comparable across hemispheres. We selected summer/winter temperature and humidity data and present details of these climate features in Supplementary Table 1. For these selected features, we implemented k-means clustering (Likas et al., 2003) to transform the data from continuous to categorical with the optimal number of clusters determined by using the R package *NbClust* (Charrad et al., 2014) as well as visual inspection. We also created a novel urbanization score for each city based on its neighboring biomes by generating a numerical score corresponding to definitions of levels of urbanization from the Anthromes Project (Ellis and Ramankutty, 2008). Anthromes are classifications of human-created ecological patterns and, in Anthromes version 1, these include dense settlements, villages, croplands, rangelands, forested areas, and wildlands. For our model, we gave a wildlands a score of 0, forested a score of 1, rangelands a score of 2, croplands a score of 3, villages a score of 4, and dense settlements a score of 5. We then added the three scores of the surrounding areas together to create an urbanization score; however, to increase predictive power in the fitted multi-output MLP model, we binarized the data and used the median urbanization score as the cutoff.

### Machine Learning

We implemented three classification algorithms, i.e., random forest (RF) (Breiman, 2001), support vector machine (SVM) (Cortes and Vapnik, 1995), and MLP (Gardner and Dorling, 1998), for the main dataset to compare the performances of different feature sets in terms of accurate city predictions. For the main dataset, we randomly selected 20% of the samples from each city and fixed these as the test set. Of the remaining samples from each city not belonging to the test set, we randomly selected 80% to serve as the training set and specified the final 20% of these remaining samples as the validation set. We repeated this procedure 100 times and recorded the error rates of the test set for each run. For each sample in the test set, we recorded the prediction results based on 100 different models. We performed RF, SVM and MLP using the R packages *randomForest* (Liaw and Wiener, 2002), *e1071* (Dimitriadou et al., 2008), and *keras* (Allaire and Chollet, 2019), respectively. For RF, we used 1,000 trees, and the count of variables chosen at each split was equivalent to the square root of the number of features in the dataset. The SVM classifier was implemented using the R function “best.svm.” The parameters in the SVM, i.e., gamma and the c-value, which affect model fitting, were obtained by testing the performance of models with different combinations of parameters. For MLP, a mixture of the rectified linear unit (ReLU) and softmax activations along with dropout (Hinton et al., 2012) were implemented.

For predicting the mystery samples, we randomly selected 20% of the mystery samples to serve as a test set for each run and then included the remaining mystery samples (labeled as “mystery”) along with the main dataset to serve as the training set. We repeated this procedure 100 times.

Since CAMDA provided non-microbial data only for cities within the main dataset, for the non-microbial city-specific data, we used the multi-output MLP to build the model, which enabled us to model the relationship between microbial features and the non-microbial city-specific features from the main dataset, where microbial features were used as the predictors and non-microbial data were used as the response. We used the model based on the main dataset to predict the non-microbial city-specific features for the mystery samples, which allowed us to draw inferences about the sample origins. In this way, we could identify information about the samples in the mystery dataset without having trained the model with labels for cities within the mystery dataset.

## Results

### Feature Selection

As discussed in the Feature Selection section in the Materials and Methods section, even after quality control and OTU picking, there were still some samples with extremely low counts compared to other samples within the same city. We removed samples with a sum of counts lower than the cutoff for the further analyses for each city in the main dataset and present the number of features before and after the filtering for each city in Supplementary Table 2. There were 1,060 and 992 samples before and after the filtering for the main dataset, respectively, with the number of filtered samples for each city ranging from 15 (Doha 2017) to 50 (New York 2017).

We performed feature selection in two steps as discussed in detail in the Feature Selection subsection in the “Materials and Methods” section and ordered the selected features by the number of times the model retained each feature; we used this ordering as cutoffs for downstream analyses. By setting different cutoffs, we obtained different feature sets.

After the first step of initial feature selection, we retained 223 features, including 35 orders, 68 families, and 120 species. For the second step of feature selection with dimension reduction with Elastic Net, we present the results with different cutoffs ranging from 6 to 14 (Supplementary Table 3). The number of features for each taxon decreases with the increase of the cutoff, particularly for the number of species.

### Clustering for the Non-microbial City Data

We performed clustering on the non-microbial city features to transform the continuous data into categorical data. Please refer to the Clustering for the non-microbial city data section of section “Materials and Methods” for further details. We present details of group assignment for each city in Table 2. Summer temperature, winter temperature, summer humidity, winter humidity, coastal indicator and neighbor biomes were categorized into 3, 4, 3, 2, 2, 2 levels, respectively, which allowed the maximum of 288 combinations of non-microbial features. For the categorical climate features, groups with higher number indicate higher values (i.e., a higher temperature will have a higher temperature score). The generated non-microbial city features were used as the response variables for the multi-output MLP.

**TABLE 2:** Details of the group assignment for each city in the main dataset.

City	Summer temperature	Winter temperature	Summer humidity	Winter humidity	Coastal indicator	Degree of urbanization of the neighboring biomes
Stockholm (ARN)	0	0	1	1	1	0
Barcelona (BCN)	1	1	1	0	1	1
Berlin (BER)	1	0	1	1	0	1
Denver (DEN)	1	0	0	0	0	0
Doha (DOH)	2	2	0	0	1	0
Fairbanks (FAI)	0	0	1	1	0	0
Hong Kong (HKG)	2	2	2	1	1	0
Seoul (ICN)	1	0	1	0	0	1
Kiev (IEV)	1	0	1	1	0	1
Ilorin (ILR)	2	3	2	0	0	0
Kuala Lumpur (KUL)	2	3	2	1	0	1
London (LCY)	0	1	1	1	0	1
Lisbon (LIS)	1	1	1	1	1	0
New York (NYC)	1	0	1	1	1	1
Offa (OFF)	2	3	2	0	0	1
Sao Paulo (SAO)	1	2	1	1	0	1
Santiago (SCL)	0	1	0	0	0	1
Sendai (SDJ)	1	0	2	1	1	1
San Francisco (SFO)	0	1	1	1	1	1
Singapore (SGP)	2	3	2	1	1	1
Taipei (TPE)	2	2	1	1	1	1
Tokyo (TYO)	2	1	1	0	1	1
Zurich (ZRH)	0	0	1	1	1	1

### Machine Learning

#### Classification Results of the Main Dataset

To find the feature set with the best classification performance, we fit the machine learning models, i.e., RF, SVM, and MLP using feature sets that were generated by using different cutoffs during feature selection. Table 3 presents the details of the classification error rate.

**TABLE 3:** Details of the classification error rates with different cutoffs.

Feature selection	Methods		
Cutoff	RF	SVM	MLP
6 (166)	0.4121	0.3618	0.3216
7 (139)	0.4271	0.3719	0.3116
8 (113)	0.4070	0.3417	0.3166
9 (86)	0.4322	0.3518	0.3367
10 (64)	0.4422	0.3618	0.3417
11 (64)	0.4372	0.3970	0.3317
12 (44)	0.4322	0.3869	0.3266
13 (35)	0.4070	0.4171	0.3417
14 (22)	0.4874	0.4824	0.4573

*The cutoff is defined as the number of times the features retained by the elastic net logistic regression.*

**FIGURE 2:**
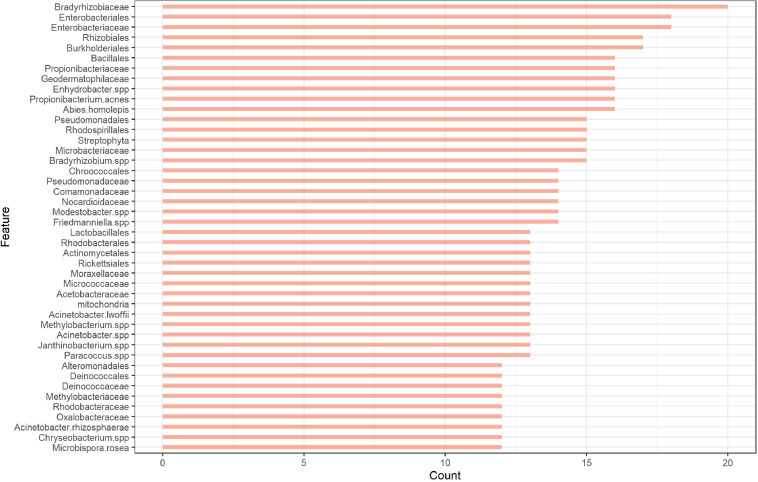
The number of times the features retained by the elastic net logistic regression.

Among the three methods, MLP showed the best performance regardless of the feature set used. Therefore, we only used MLP for the mystery samples’ prediction. For the MLP, the lowest error rate was obtained when the feature set with cutoff = 7 was used. A cutoff of 7 indicates that the feature was retained by the fitted elastic net in at least 7 of the 28 fitted elastic net models. However, we noticed that a comparable error rate was obtained without including too many features when we used a cutoff of 12 for the feature set. The feature set with cutoff = 12 was therefore preferred for further analysis since it better balanced error rate and number of included features. The OTU count data with selected features (cutoff = 12) for the samples that passed quality control in the main and mystery datasets can also be found in the Supplementary Material for this paper. This feature set consisted of 44 features including 14 orders, 16 families and 14 species. The counts of features retained by the elastic net models are presented in Figure 2. Among the 44 features, the top 5 features that were retained by the models the highest number of times were the families *Bradyrhizobiaceae* and *Enterobacteriaceae* and the orders *Enterobacteriales*, *Burkholderiales*, and *Rhizobiales. Bradyrhizobiaceae* and *Enterobacteriaceae* are families with the corresponding orders *Rhizobiales* and *Enterobacteriales*, respectively, and *Enterobacteriales* contains only the *Enterobacteriaceae* family (Jenkins et al., 2017). The *Bradyrhizobiaceae* family consists of 11 diverse genera; the organisms belonging to this family include nitrogen fixing bacteria, photosynthesizing bacteria, bacteria which use anaerobic and/or aerobic respiration, organisms involved in the sulfur cycle, and human pathogens (the pathogen which causes cat-scratch fever belongs to this family) (De Souza et al., 2014). Many of the members of the *Enterobacteriaceae* family, including the genera *Escherichia*, *Salmonella*, *Shigella*, *Klebsiella*, and *Serratia*, live in the intestinal tracts of humans and animals; some of these are regular species in normal microbiota while others can cause disease in humans (Rock and Donnenberg, 2014). The order *Burkholderiales* includes many genera that cause disease in humans and many bacteria that occur naturally in the environment (Wang et al., 2018). Finally, the order *Rhizobiales* interact symbiotically with plants and can provide various nutrients, phytohormones, and precursors for plant metabolites; it contains many genera of nitrogen-fixing, methanotrophic, legume-nodulating, microsymbiotic bacteria (Erlacher et al., 2015). From this, one can see that the most valuable features are associated with bacteria that commonly live in or on humans or bacteria that correspond to the surrounding environment.

**FIGURE 3:**
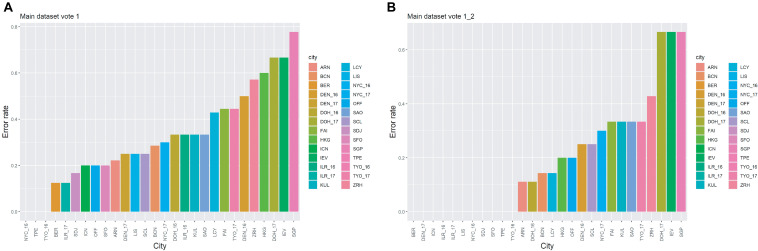
The error rates for the cities in the main dataset. **(A)** The results are based on the highest vote. **(B)** The results are based on the highest and the second-highest votes.

When using the feature set with cutoff = 12 for MLP (the classifier with the highest vote of the prediction results from the 100 runs), we found that the error rate was 32.66%. However, for some samples, the highest vote and the second-highest vote tied. Furthermore, some of the second-highest votes were the true label for the samples. When the highest and second-highest votes were used at the same time, the error rate fell to 19.60%, indicating that nearly 13% of the second-highest vote of the samples were the true labels. The details of the error rate for each city are presented in Figure 3. As seen from Figure 3A, there are three cities with error rates = 0; these are New York (2016), Taipei and Tokyo (2016). When the second-highest vote was used also, the number of the cities with error rates = 0 increased to 11 (Figure 3B). Additionally, as samples collected from the same city but in different years were regarded as samples from different cities in this work, e.g., NYC_16 and NYC_17 in Figure 3, it was worth investigating whether the year of prediction for these samples was ever reversed. By looking into the details of the results of 100 runs for the test set, we found that among the samples from the cities with multiple collecting years, only 2 out of 68 samples were predicted to be the same city with a different collection year, indicating the possibility of technical variability within the samples due to batch effects even though the samples were collected from the same city. This corresponds to the results in Figure 3, which shows that the error rates for samples from the same city but collected in different years could be vastly different, e.g., TYO_16 and TYO_17, DOH_16 and DOH_17. In addition, the difference in the error rates for the samples from the same city with different years could be due to unavoidable, dynamic changes within the city’s microbial community. However, due to unavailability of data across multiple years for all cities, it was not possible to estimate the confounding factor.

#### Prediction Results for the Mystery Dataset

Next, we implemented the same procedure to predict the non-microbial data of the test set by using a multi-output MLP with the selected feature set. The only difference was that we combined samples from the same city collected in different years to predict the non-microbial data, which we then used separately for predicting the sample origins. The error rates were 20.1, 28.6, 24.6, 21.1, 15.6, and 14.1%, respectively, for summer temperature, winter temperature, summer humidity, winter humidity, coastal indicator, and degree of urbanization of the neighboring biomes.

Finally, we used the selected feature set to predict the sample origins and the non-microbial features for the mystery samples. There were samples from 10 cities in the mystery dataset, consisting of five cities that have been sampled before and five new cities. These were Bogota, Hong Kong, Kiev, Krakow, Marseille, Naples, Taipei, Tokyo, Vienna, and Zurich. For the samples from the cities that have been sampled before, the prediction was straightforward; we could predict the sample origins directly. The error rates for these five cities, i.e., Hong Kong, Kiev, Taipei, Tokyo, and Zurich, based on the highest and second highest votes were 46.7, 72.7, 18.18, 38.5, and 50%, respectively. The lowest error rate was obtained from Taipei, which showed the best performance when using only data from the main dataset as well. Similarly, the samples from Kiev had the highest prediction error rate for both the main dataset and the mystery dataset among these five cities. For the samples from the cities that do not appear in the main dataset, we used the classifier to see if we could determine whether the test samples were accurately labeled “mystery” (for more details, please refer to the subsection Machine Learning in the “Materials and Methods” section). 57.7% of the samples were predicted not to belong to a city in the main dataset (“mystery”). The knowledge of whether the samples have been sampled before provided limited information, so we then predicted a selection of non-microbial features for the samples from the new cities. The average predicted non-microbial features for each city are presented in Table 4. Based on the results, we found that the pattern of Krakow was similar to the pattern of Berlin in the main dataset, and the pattern of Naples was similar to the pattern of New York in the main dataset. According to the geographic location, Krakow, Poland is close to Berlin, Germany; Naples, Italy and New York City, United States are nearly at the same latitude. This additional information could help us to narrow the range of possible cities for predicting the mystery sample origins. However, for the other three cities, we found the average of some features were not close to the preset level, and some were even not in accordance with reality. For example, the average summer temperature score of the samples from Bogota was 1.417, indicating that the prediction for this feature was different for diverse samples as some of the samples were predicted to level 1 while other samples were predicted to level 2. Marseille’s coastal indicator, Vienna’s summer temperature, and Vienna’s coastal indicator also exhibited similar behavior. Additionally, according to the results in Table 4, the winter temperature of Bogota was predicted as being close to level 0; however, the true winter temperature of Bogota should correspond to the higher level. The misclassification of the samples could be caused by the variability of the samples within the same city. This happened in the 2019 CAMDA challenge as well; some samples cannot be classified correctly by any of the machine learning methods we used, which meant that the microbial composition of these outlier samples could be different from the other samples in the same city, making them difficult to identify accurately. Furthermore, the samples from the novel origins may not be fully represented by the features selected based on the main dataset, resulting in the failure of accurately predicting some of the samples.

**TABLE 4:** The average predicted level for non-microbial features for new cities in the mystery dataset.

City	Summer temperature	Winter temperature	Summer humidity	Winter humidity	Coastal indicator	Degree of urbanization of the neighboring biomes
Bogota	1.417	0.25	1	0.917	0.833	0.917
Krakow	1	0	0.909	0.909	0.091	0.909
Marseille	0.9	0.2	1.1	0.9	0.4	0.9
Naples	1	0	1	1	0.889	0.889
Vienna	0.6	0	1	1	0.4	0.8

## Discussion and Conclusion

In this work, we have used a more relaxed preliminary filtration to obtain the common features and followed with an elastic net logistic regression for further selection. Of the three different classification methods we tested, we found that MLP performed the best of the three regardless of the feature sets. For MLP, we obtained a low error rate without including too many features by using the feature set with cutoff = 12. The top five features were common orders and families, which corresponded with our results from the CAMDA 2019 MetaSUB challenge. The error rate was 19.60% based on the highest and the second-highest votes. Compared to last year’s challenge, there were many more samples, with over 1,000 samples included. However, higher error rates were obtained since we included more cities this year, which made the accurate prediction of cities harder. In addition, we found that samples collected from the same city but collected over different years sometimes displayed notably different error rates, including NYC_16 and NYC_17, TYO_16 and TYO_17, and DOH_16 and DOH_17. This could be caused by technical variability such as batch effects. The prediction accuracy could be improved by a better normalization method to avoid the potential batch effects. Furthermore, this difference between samples collected in different years could also be considered as the result of dynamic change in microbial communities within a given city from year to year.

According to the results for predicting the mystery samples, Taipei has the best accuracy while samples from Kiev were poorly classified. For the samples of cities that do not exist in the main dataset, we found that the pattern of the samples from Krakow was similar to the pattern of Berlin, and the pattern of the samples from Naples was similar to the pattern of New York. These results made sense to us since Krakow and Berlin are geographically close, and Naples and New York are at nearly the same latitude. This additional information would help us to draw inferences and narrow the possible candidates for the sample origins. Since we did not have the non-microbial city-specific data for the mystery cities, we were unable to validate the error. The predictions for some novel cities including Bogota, Marseille, and Vienna were not ideal; some of the non-microbial city features predictions were not close enough to the preset level (Table 4), indicating the variability of the microbial composition of samples within the same cities. One of the drawbacks of the multi-output MLP is that it cannot provide labels for combinations it has not seen. For this challenge, the non-microbial city features were only provided for cities in the main dataset; therefore, the pattern of the samples predicted by the model could be only mapped to the cities we already had in our dataset. Compared to the microbial data, the non-microbial data of different cities are easy to collect and are easily accessible; many of these were also readily available online. The prediction could be improved with the inclusion of data from more cities.

The average of the error rates of the six non-microbial city-specific features was about 20%; it was necessary to select meaningful and appropriate features to decrease the error rate and thus allow us to build a more reliable model. Currently, the selection of the non-microbial features was based on our knowledge of the factors. We have found that some features such as climate may affect the microbial composition, which motivated us to use the microbial features to inversely predict these non-microbial features and further obtain the city’s pattern. In addition to the literature support, other analyses to determining the main drivers of variance in microbial features would also be worth investigating in future analyses.

To date, our work has been based on generating taxonomic information from alignments of the reads to the 16S ribosomal gene in bacterial species (Walker et al., 2018; Walker and Datta, 2019; Zhang et al., 2021). This is a limitation considering that the DNA samples also contained DNA from microorganisms that are not bacteria. By mapping the reads not only to a limited bacterial genome region but to a much wider range of full genomes from many other types of microorganisms including fungal, viral, and eukaryotic species, new information will be added to the machine learning algorithms and will likely enable them to model geographic regions more accurately. We are working on approaches to achieve this goal, and our preliminary work has yielded interesting results with counts from a variety of microbial species that are not captured by 16S sequencing.

In summary, the results presented in this work show an effective method to process and classify microbial samples by origin. Even when samples were from cities that have never been sampled before, we could map these samples to a specific pattern of climate information and draw inferences from these predictions. By doing this, it would be possible for us to obtain the trace of the object based on the swabs taken from the object. However, there is still much to be improved in our future work.

## Data Availability Statement

Publicly available datasets were analyzed in this study. This data can be found here: http://camda2020.bioinf.jku.at/doku.php/contest_dataset.

## Author Contributions

SD reviewed the manuscript and provided the theoretical support when required. RZ, DE, and AW designed and ran the analyses. RZ wrote the manuscript. All authors have read and approved the final manuscript.

## Conflict of Interest

The authors declare that the research was conducted in the absence of any commercial or financial relationships that could be construed as a potential conflict of interest.

## Publisher’s Note

All claims expressed in this article are solely those of the authors and do not necessarily represent those of their affiliated organizations, or those of the publisher, the editors and the reviewers. Any product that may be evaluated in this article, or claim that may be made by its manufacturer, is not guaranteed or endorsed by the publisher.

## Footnotes

1
http://camda2020.camda.info/
