# Supplementary material for: Unraveling City-Specific Microbial Signatures and Identifying Sample Origins for the Data From CAMDA 2020 Metagenomic Geolocation Challenge
Source: Front Genet. 2021 Aug 5;12:659650. doi: 10.3389/fgene.2021.659650 (PMC8375386; doi:10.3389/fgene.2021.659650)
Supplement: Supplementary file 1 [file Data_Sheet_1.zip › Supplementary Material/Supplementary_Material.docx]

Supplementary Material

# Supplementary Data

The following files contain OTU (operational taxonomic units) counts data for the main and mystery dataset with the selected features (cutoff = 12) and qualified samples.

**Main_count.csv:**

OTU counts data for the main dataset with the selected features (cutoff = 12) and qualified samples.

**Mystery_count.csv:**

OTU counts data for the mystery dataset with the selected features (cutoff = 12) and qualified samples.

The following files contain metadata associated with the samples.

**Meta_mystery.csv:**

Metadata for mystery samples.

**weather_meta.csv:**

Weather related metadata.

**location_meta.csv:**

Location related metadata.

**biomes_meta.csv:**

Biomes related metadata.

# Supplementary Figures and Tables

## Supplementary Tables

**Table S1.** Details of the selected climate features for the clustering.

| Climate-related | Month | Statistics | Categorical |
| --- | --- | --- | --- |
| Temperature | Summer_month_1 | Maximum | Summer_temperature |
|  |  | Average |  |
|  | Summer_month_2 | Maximum |  |
|  |  | Average |  |
|  | Summer_month_3 | Maximum |  |
|  |  | Average |  |
|  | Winter_month_1 | Average | Winter_temperature |
|  |  | Minimum |  |
|  | Winter_month_2 | Average |  |
|  |  | Minimum |  |
|  | Winter_month_3 | Average |  |
|  |  | Minimum |  |
| Humidity | Summer_month_1 | Maximum | Summer_humidity |
|  |  | Average |  |
|  |  | Minimum |  |
|  | Summer_month_2 | Maximum |  |
|  |  | Average |  |
|  |  | Minimum |  |
|  | Summer_month_3 | Maximum |  |
|  |  | Average |  |
|  |  | Minimum |  |
|  | Winter_month_1 | Maximum | Winter_humidity |
|  |  | Average |  |
|  |  | Minimum |  |
|  | Winter_month_2 | Maximum |  |
|  |  | Average |  |
|  |  | Minimum |  |
|  | Winter_month_3 | Maximum |  |
|  |  | Average |  |
|  |  | Minimum |  |

**Table S2.** Number of samples before and after the filtering for each city in the main dataset.

| City | Year | Number of Samples | Number of filtered samples |
| --- | --- | --- | --- |
| Stockholm (ARN) | 2017 | 50 | 47 |
| Barcelona (BCN) | 2016 | 38 | 36 |
| Berlin (BER) | 2016 | 41 | 41 |
| Denver (DEN) | 2016 | 23 | 20 |
|  | 2017 | 22 | 21 |
| Doha (DOH) | 2016 | 48 | 47 |
|  | 2017 | 15 | 15 |
| Fairbanks (FAI) | 2016 | 48 | 44 |
| Hong Kong (HKG) | 2017 | 49 | 48 |
| Seoul (ICN) | 2017 | 50 | 48 |
| Kiev (IEV) | 2017 | 49 | 43 |
| Ilorin (ILR) | 2016 | 47 | 47 |
|  | 2017 | 50 | 41 |
| Kuala Lumpur (KUL) | 2017 | 30 | 30 |
| London (LCY) | 2017 | 37 | 33 |
| Lisbon (LIS) | 2016 | 19 | 19 |
| New York (NYC) | 2016 | 49 | 35 |
|  | 2017 | 50 | 50 |
| Offa (OFF) | 2016 | 26 | 26 |
| Sao Paulo (SAO) | 2017 | 29 | 29 |
| Santiago (SCL) | 2016 | 26 | 21 |
| Sendai (SDJ) | 2017 | 32 | 30 |
| San Francisco (SFO) | 2017 | 27 | 25 |
| Singapore (SGP) | 2017 | 48 | 47 |
| Taipei (TPE) | 2017 | 50 | 49 |
| Tokyo (TYO) | 2016 | 25 | 23 |
|  | 2017 | 49 | 44 |
| Zurich (ZRH) | 2017 | 33 | 33 |
| All cities | - | 1060 | 992 |

**Table S3.** The number of features for different cutoffs. The cutoff is defined as the number of times the features retained by the elastic net logistic regression.

| Cutoff | Species | Family | Order |
| --- | --- | --- | --- |
| 6 | 78 | 57 | 30 |
| 7 | 64 | 48 | 27 |
| 8 | 51 | 37 | 25 |
| 9 | 35 | 31 | 20 |
| 10 | 27 | 22 | 15 |
| 11 | 17 | 17 | 15 |
| 12 | 14 | 16 | 14 |
| 13 | 11 | 12 | 12 |
| 14 | 6 | 8 | 8 |
